# Rare Case of Osseus Metaplasia in a Detached Remnant Leiomyoma Following Hysteroscopic Myomectomy

**DOI:** 10.7759/cureus.24156

**Published:** 2022-04-15

**Authors:** Maha Al Jumaily, Emad Mikhail

**Affiliations:** 1 Obstetrics and Gynecology, University of South Florida Morsani College of Medicine, Tampa, USA

**Keywords:** myomectomy, uterine leiomyoma, leiomyoma, ob-gyn, gyn pathology, hysteroscopic polypectomy/myomectomy, osseus metaplasia, myoma uteri

## Abstract

Uterine leiomyoma is the most common benign tumor of the uterus, affecting reproductive-age women. Although women with uterine fibroids are commonly asymptomatic, in symptomatic patients, hysteroscopic myomectomy is considered the first-line surgical treatment for intracavitary fibroids in women who wish to maintain fertility.^ ^

Osseous metaplasia in uterine fibroids is the transformation of fibroids cells into pure mature or immature bone. It is rare, and few case reports present with osseous metaplasia in uterine fibroids. This is the first report in the literature of osseous metaplasia in a remnant fibroid after hysteroscopic myomectomy. Every effort should be attempted to ensure complete retrieval of the detached fibroid remnant after hysteroscopic resection, as this might decrease the risk for subsequent surgeries.

## Introduction

Uterine leiomyoma is the most common benign tumor of the uterus, affecting reproductive age women with a prevalence of 68.8% [1-3] Although most women with uterine fibroids are asymptomatic [4], the symptoms might include heavy and prolonged menstrual periods, pelvic pain and pressure, dyspareunia, bladder symptoms, abnormal bowel movements, and infertility [1].

Symptoms of uterine fibroids can be managed medically or surgically, and many factors determine that type of treatment, like the severity of the symptoms, size and location of the fibroids, and the desire to preserve fertility. Hysteroscopic myomectomy is considered a first-line surgical treatment for intracavitary fibroids in women who wish to preserve fertility [5].

Osseous metaplasia in leiomyomas is a rare condition characterized by the transformation of the fibroid tissue into osteoblast (immature or mature bone). We present a case report for a patient with a remnant uterine leiomyoma that transformed into the bone after a prior hysteroscopic myomectomy.

## Case presentation

A 37-year-old female, G1P0010, presented to our clinic complaining of infertility for the past year. She underwent hysteroscopic myomectomy to treat symptomatic submucosal uterine fibroid. She was evaluated by a reproductive endocrinology and infertility colleague and diagnosed with a remnant submucosal myoma; the patient desired surgical removal.

**Figure 1:**
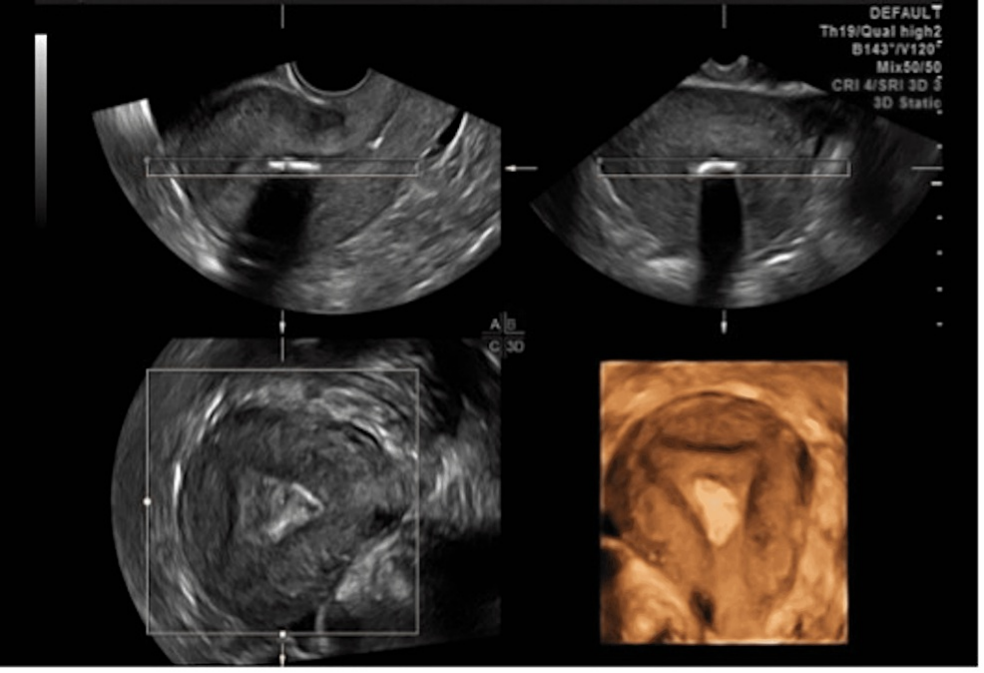
Transvaginal ultrasound image with 3D construction showing the submucosal location of the fibroid

Transvaginal ultrasound showed two small uterine fibroids (Figure 1). The first uterine fibroid measured 19 mm x 6 mm x 13 mm, type 0, with a calcified, pedunculated intracavitary hyperechoic lesion. The other one was type 5, subserosal with more than 50% intramural and measured 12 mm x 8 mm x 14 mm.

**Figure 2:**
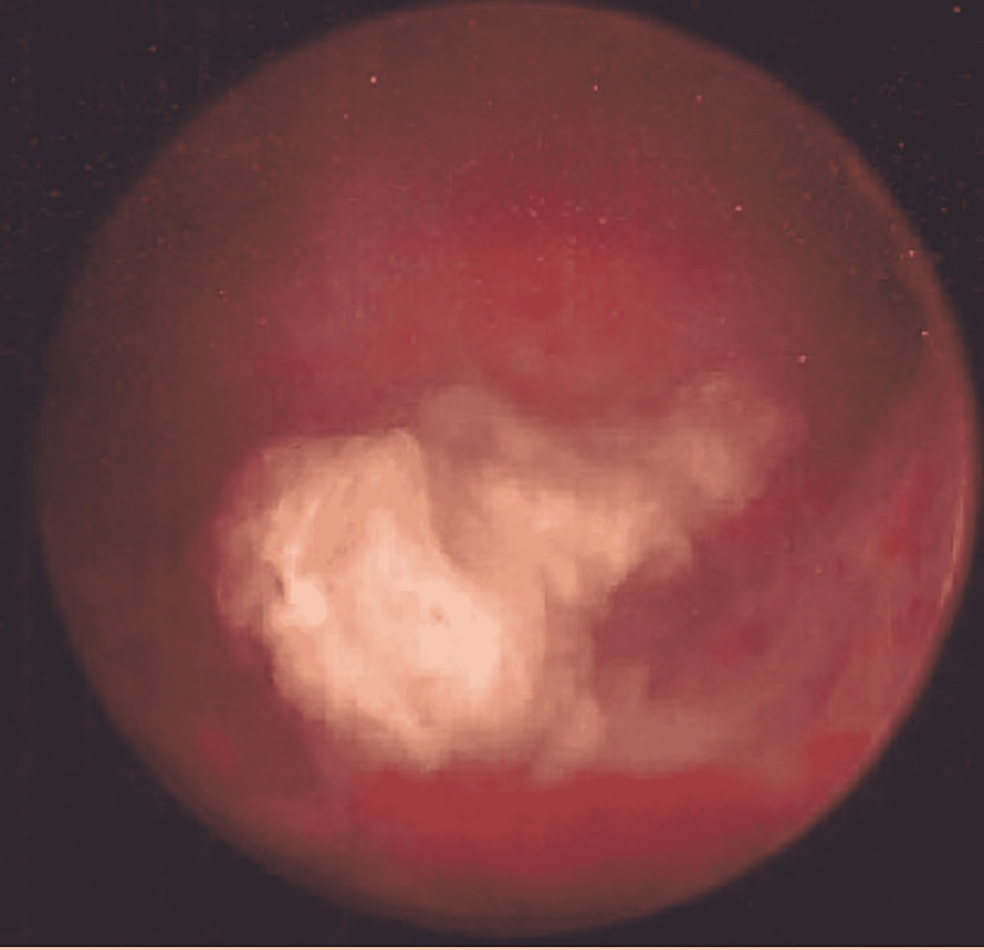
Hysteroscopic view showing the calcified fibroid remnant

**Figure 3:**
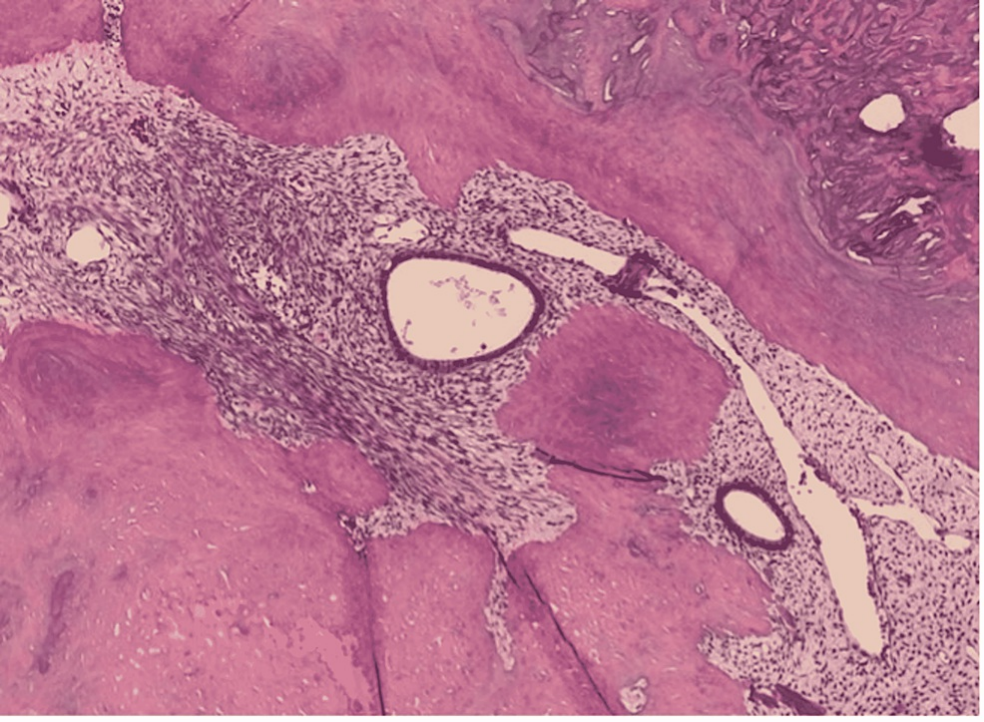
A microscopic slide showing the osseous metaplasia

The patient underwent a repeat hysteroscopic myomectomy to remove the submucosal uterine leiomyoma. Intraoperative findings included a 3 cm long strip of fibroid remnant, free-floating inside the uterine cavity (Figure 2). Surgical removal was attempted using a hysteroscopic morcellator but was not successful as the tissue was very calcified. Cervical dilation followed by extraction using hysteroscopic grasper was performed. Surgical pathology revealed calcified endometrial fibroid and irregular bone with entrapped endometrial stroma consistent with osseous metaplasia (Figure 3).

## Discussion

Uterine leiomyomas are a common nonmalignant tumor of women during their reproductive age [6]. It is more common among African- American women [7]. Although the etiology of the uterine fibroids is still unclear, there are some factors related to their growth and development, such as age, genetics, hormonal factors (estrogen and progesterone), steroids, and micro-RNAs (miRNAs), cytokines and chemokines [6,8]. Diagnosis of uterine fibroids is usually based on clinical symptoms, physical examination, and imaging such as pelvic ultrasound. The most common symptom is abnormal uterine bleeding, such as metrorrhagia or menorrhagia. Other symptoms include anemia, pelvic pain, dyspareunia, urinary symptoms such as urgency or incontinence, and bowel symptoms such as constipation [9].

Uterine leiomyoma contains extracellular matrix and cells with a low mitotic index [10]. Classification of uterine fibroids depends on their location within the uterus and are classified into submucosal (SM), intramural (IM), and subserosal (SS) [11]. Uterine fibroids can undergo degeneration and become calcified. Calcified fibroids occur in 8% of cases and can be seen at any age, but they are more common in postmenopausal women [12]. Osseous metaplasia in uterine fibroids is the transformation of fibroids cells into pure mature or immature bone. It is rare, and few case reports present with osseous metaplasia in uterine fibroids. This is the first report in the literature of osseous metaplasia in a remnant fibroid after hysteroscopic myomectomy. The etiology of osseous metaplasia in uterine fibroids is still unclear; however, calcification and chronic inflammation play a major role in the pathophysiology process [12].

Where possible, hysteroscopic myomectomy is preferred because of its efficacy and reduced surgical morbidity [13]. Complete resection of the submucosal myoma should be attempted as much as possible. Incomplete resection can be subdivided into incomplete tissue resection versus incomplete tissue retrieval (as in the case presented here). Complete resection decreases the need for subsequent surgery [14]. Incomplete resection is associated with an increased risk factor for repeat surgery in the future, with half of the patients with incomplete resection requiring additional surgery for recurrence within two years [15].

Historically, by far the most common hysteroscopic technique has been transcervical resectoscope myomectomy (TCRM) with a resectoscope, first reported in 1976. However, there now exists a growing number of other hysteroscopic techniques for dissection, vaporization, or morcellation and excision of submucosal myomas [13].

In cases where resections have been performed with monopolar energy devices, reaching fluid deficit limit is the main reason for incomplete resection [14], which could be the reason why the fibroid remnant was left inside.

## Conclusions

Osseous metaplasia of a detached remnant uterine leiomyoma is a rare presentation with an unclear etiology. The complete retrieval of the detached remnant might decrease the risk for subsequent surgeries. Retrieval of leiomyoma fragments is intuitive in cases where hysteroscopic morcellators are utilized. In patients where resectoscopic surgery is indicated, a thorough evaluation of the cavity and meticulous retrieval of fragments is important to avoid such a complication.
